# The Adolescent's Competency for Interacting with Alcohol as a Determinant of Intake: The Role of Self-Regulation

**DOI:** 10.3389/fpsyg.2017.01800

**Published:** 2017-10-26

**Authors:** Jesús de la Fuente, Inmaculada Cubero, Mari Carmen Sánchez-Amate, Francisco J. Peralta, Angélica Garzón, Javier Fiz Pérez

**Affiliations:** ^1^School of Psychology, University of Almería, Almería, Spain; ^2^Department of Psychology, Facultad de Ciencias Sociales y Humanidades, Universidad Autónoma de Chile, Santiago de Chile, Chile; ^3^School of Psychology, Fundación Universitaria Konrad Lorenz, Bogotá, Colombia; ^4^Department of Psychology, Universita Europea di Rome, Rome, Italy

**Keywords:** interacting with alcohol competency, self-regulation, attitudes, adolescence, prevention program

## Abstract

The competency for interacting with alcohol is a highly useful Educational Psychology model for preventing and for understanding the different behavioral levels of this interaction. Knowledge of facts, concepts and principles about alcohol use, self-regulated behavior, and attitudes toward alcohol are predictive of adequate interaction with alcohol. The objective of this study was to empirically evaluate this postulated relationship. A total of 328 Spanish adolescents participated, between the ages of 12 and 17. All were enrolled in 1st–4th year of compulsory secondary education, in the context of the ALADO Program for prevention of alcohol intake in adolescents. An ex post facto design was used, with inferential analyses and SEM analyses. Results show an interdependence relationship, with significant structural prediction between the behavioral levels defined and the level of alcohol intake, with principles, self-regulating control and attitudes carrying more weight. Analyses are presented, as are implications for psychoeducational intervention using preventive programs based on this competency model.

## Introduction

Alcohol abuse in adolescents is an old problem (Cortés et al., 2007; Farke and Anderson, 2007; Castellanos-Ryan et al., 2013; Chassin, 2015). The problem stems in part from the firmly entrenched role of alcohol in Western culture. Whether we like it or not, alcohol use is a culturally institutionalized habit in adult social relations; the adolescent simply imitates this. In the period of transition of childhood to adulthood, alcohol use with one's friends is part of the initiation rite in our culture (Ballester et al., 2000; Espada et al., 2008). Social pressure in the case of alcohol is very strong in all spheres of the community, in day-to-day relationships around town, and even in work relationships (Dodge et al., 2009; Zaldívar et al., 2011; Trucco et al., 2014).

In Spain, according to the Ministry of Health and Consumption (Ministerio de Sanidad y Consumo, 2005), adolescents beginning at puberty are more willing to engage in risk behaviors such as alcohol use, and at the same time, their relationship to the school context and even their academic achievement are more likely to decline (Bermúdez et al., 2009). Later, a study carried out by the *Spanish Observatory on Drugs and Drug Addiction* (Ministerio de Sanidad, Servicios Sociales e Igualdad, 2016; Observatorio Español sobre Drogas, 2017) stated that during 2014, 78.9% of secondary school students between the ages of 14 and 18 years had consumed alcohol regularly during the past month, and initial alcohol intake was established at an average age of 13.8 years. Similarly, the percentage of students who had experienced acute alcohol intoxication in the past month was 33.1%, and binge drinking was practiced by 47.3%.

There is consensus that no one personality type predisposes toward alcoholism, but there are certain important characteristics common to all adolescents who abuse alcoholic drinks, namely, extraversion, nervousness and lack of control. Many studies that have addressed the topic of personality and addiction conclude that some of these characteristics are linked to addictive behavior (Fantín, 2006, 2007; Calvete and Estévez, 2009). Behavior patterns from childhood tend to continue into adolescence; if these habits are inadequate, fewer healthy behaviors and more risk behaviors are produced during adolescence and beyond. Likewise, as age increases, poorer personal adjustment is observed. Stress is a risk factor associated with drug use, and the idiosyncrasies of adolescence involve an increase in stress, something that many studies have agreed on (Windie and Windie, 1996; Nadal, 2008).

There is a great deal of current research on this topic, given that consumption of alcoholic beverages has become popular among adolescents and the age of initiation has gone down (Plan nacional sobre Drogas, 2003). Moreover, when addressing this topic, all related factors must be taken into account (Senra, 2003). The effects of alcohol and drug use during early and mid-adolescence are a real cause for concern, and are related to health problems (Bento et al., 2013), problems at school (Ekberg et al., 2016) mental disorders (Borges et al., 2017), unprotected sex (Boyer et al., 2017), and delinquency (Doherty et al., 2008). As in most Western countries, alcohol use among adolescents in Spain is very high. All this data is evidence of the need to take preventive measures in the spheres of family, school and society (Villatoro et al., 2002).

### Limitations of the classic models for preventing alcohol intake

Taking the assumptions of a *medical approach* to primary prevention, traditional strategies for preventing alcohol intake have focused mainly on equipping adolescents with information (Ballesteros et al., 2003). From the *bio-psycho-social approach*, prior evidence has pointed out the limitations of such an approach, as well as the need to use comprehensive training programs that include self-improvement skills and social skills (Marlatt and Witkiewitz, 2002; De Ridder and de Wit, 2006; Lemstra et al., 2010). Some recent studies from experimental and clinical approaches have reported improvement effects in the adolescents' psychological variables, such as self-esteem and self-efficacy (Alexander and Anderson, 2017), internalizing personality variables (anxiety) and externalizing variables (behavior problems) (Scalco et al., 2014; O'Leary-Barrett et al., 2016; Wills et al., 2016). The role of social identity has also been analyzed, through online contexts (Pegg et al., 2017), and universal programs for preventing alcohol intake in adolescents have been applied (Teesson et al., 2017). Some improvement is seen in all cases, but without conclusive results.

An interactive approach from Educational Psychology, the recent Theory of Self- vs. Externally-Regulated Learning (SRL vs ERL) (de la Fuente, 2015, 2017), has focused on the nature of the adolescent (Oliva et al., 2002; Oliva, 2007), and his/her environment–whether regulatory, a-regulatory or dysregulatory. Regulation is assumed to be an essential procedural component of the competency for managing alcohol intake and other chronic health concerns (Clark et al., 2001; Hull and Slone, 2004; Duell et al., 2016). Thus, the worst scenario for primary prevention would consist of an adolescent with a low level of self-regulation, and a profile of dysregulatory behavior (negative proactivity toward alcohol intake) in interaction with a dysregulatory context (actively encouraging consumption), thereby establishing a high probability of consumption and nonadherence to preventive treatments. In accordance with this theory, the *competency model* incorporates different behavioral levels in a wholistic fashion, and serves as a model of an *Educational Psychology intervention*, being distinct from other approaches, as has been illustrated in the case of academic stress (de la Fuente, 2015; de la Fuente et al., 2017).

### The interacting with alcohol competency, for preventing early alcohol intake

An individual may be considered *competent* when, in a given context, he or she is able to effectively and efficiently solve the problems that arise (Roe, 2002, 2003). From an Educational Psychology point of view, other authors have established that being competent means having the knowledge, skills and attitudes that allow a person to make an appropriate response to a particular, real, problematic situation (de la Fuente et al., 2005). This gives rise to a three-fold division into subcompetencies that are necessary in order to be competent in any field of knowledge: *conceptual* (knowing), *procedural* (being able to, knowing how) and *attitudinal* (wanting to, being). In the specific case of educational prevention of alcohol intake, this approach means delimiting the level of subcompetencies that adolescents possess at each level.

1. Regarding the *conceptual subcompetency:* adolescents' knowledge and their perception of the risk involved with alcohol is different from that of adults, and this in itself is a factor that predisposes toward some dangerous or inadequate behavior. All epidemiological studies agree that adolescent alcohol use in our day is abusive, and the authors conclude that this comes from adolescents' not understanding the real risks. For this reason it is very important to collect their opinions and what they conceive to be facts, concepts and principles in regard to alcohol. According to earlier research studies, adolescents' knowledge and principles center on reasons to justify the use of alcohol and the consequences of alcohol use (Arias et al., 2007).
2. Regarding the *procedural subcompetency:* personal *self-regulation* is an essential meta-skill for managing behavior. de la Fuente et al. (2009) have established the hypothesis of lack of personal self-regulation as a variable that determines alcohol intake in adolescents. The structural factors of personality and self-regulation, among others, are related to prosociality. Difficulties in exercising self-control may be risk factors for drug use (Barry et al., 2007; Calvete, 2008; O'Connor and Colder, 2015). Cognitive schemas with insufficient self-control are associated with drug use, and this cognitive style, where limits and tolerance to frustration are lacking, is quite common in our present-day environment (Urra, 2006). Adolescents who do not have adequate self-regulation do not usually plan their behavior, they have no fixed goals, nor do they monitor to what extent their behavior would move them toward such goals. Rather, they act impulsively, with disturbing results in both the academic and personal/social spheres (Eisenberg et al., 1996; King et al., 2013). A large number of studies on the importance of self-regulation have focused on addictive disorders linked to gambling and substance abuse (Madden et al., 1997; Hull and Slone, 2004), especially to alcohol use (Brown et al., 1999; Carey et al., 2004; Pearson et al., 2013). These effects are especially relevant in adolescence and youth—two stages of development that are characterized by a search for personal identity, moving away from the family context and connecting to one's peer group. Consequently, self-regulation might act as an indicator of adolescents' resilience in situations of greater psychosocial risk (Artuch-Garde et al., 2017).
3. Regarding the *attitudinal subcompetency. Attitudes, values* and *habits* toward alcohol intake have been shown to be powerful predictors of adolescents' alcohol intake behavior. Evidence has shown significant relationships between *attitudes* and *values* toward alcohol (Moral et al., 2004; Moreno, 2006). Especially important for their value in predicting behavior are alcohol intake *habits* (Mora-Ríos and Natera, 2006; Rodrigo et al., 2006). See Chart 1.

**Chart 1:** Multidimensional nature of the The Interacting with Alcohol Competency model (ALADO Program; de la Fuente et al., 2012).

1) *Knowing*:	*Facts:* knowledge of the everyday facts and uses of alcohol: problems, uses and abuses, adolescent consumption data^*^. *Concepts:* concept of alcoholism, biological and neurological effects of alcohol on the adolescent brain^*^. *Principles:* rules of use, responsible fun, respect for one's own body^*^.
+	
2) *Know how*: (Skills)	*Instrumental skills*: social skills. *Meta-behavioral skills* for managing stress: self-regulation strategies^*^.
+	
3) *Mindset* (Attitudes)	*Attitudes and values*: behavioral confidence, self-esteem, self- efficacy, value of abstinence^*^. Study habits, sport habits, fun habits, free time habits.

* *Variables in this research*.

### Adjustment in interacting with alcohol

The concept of *adjustment in interacting with alcohol* is understood to be modulated by age, context and culture. In adolescence, this adjustment can be conceived as the behavior of delaying contact with alcohol in a *proactive regulatory* manner (de la Fuente, 2017), that is, voluntary nonconsumption of alcohol and substituting it with other more adaptive behaviors, such as sports, leisure activities or having fun without recurring to harmful substances. In other words, adopting the absence of substance use in order to gain a state of *bio-psycho-social wellbeing* (Becoña, 2007a,b).

### Objectives and hypotheses

This research study has several motivations. On one hand, prior research on alcohol prevention has addressed many variables from a partial standpoint, seeking to establish probabilistic relationships between isolated variables and alcohol use in adolescents. However, an Educational Psychology approach, adopting the conceptions of competency and subcompetency, would seek to integrate the different variables into a more powerful schema. Based on the foregoing, then, the objectives and hypotheses of this study were as follows:

1. To determine any interdependence relationships between the low-medium-high level of each subcompetency with respect to the other subcompetencies, and to adjustment in interacting with alcohol. Consequently, *Hypothesis 1* established that the low-medium-high level of *conceptual* knowledge (facts, concepts and principles, especially the latter), *procedural* knowledge (self-regulation, with self-control having greater weight) and *attitudinal* knowledge (values about alcohol intake) will be mutually determined. Finally, they will determine the level of adjustment in interacting with alcohol.
2. To establish any structural predictive relationships between the different subcompetencies. In this case, *Hypothesis 2* established that adjustment in interacting with alcohol (nonconsumption) will be determined by conceptual type factors (more principles than facts/concepts), procedural type (self-regulation, especially level of control) and attitudinal type, although the latter are mediated by procedural variables.

## Methods

### Participants

The population under study were students from public secondary schools in a southern province of Andalusia (Spain). In order to include different types of schools in this investigation, schools were selected from three types of urban areas: (1) center of town, from a medium-high social stratum (*n* = 106), (2) surrounding neighborhoods, from a medium-low social stratum (*n* = 101) and (3) outlying, marginalized population areas with compensatory education, from a low social stratum (*n* = 121). The homeroom teachers from every group participated voluntarily in the experiment, having been invited by the School Psychology adviser of the local Teacher Development Center. Participating students were between the ages of 12 and 17 years [12 (*n* = 51), 13 (*n* = 80), 14 (*n* = 97), 15 (*n* = 55), 16 (*n* = 35), and 17 (*n* = 10)] and were enrolled in compulsory secondary education (grades 7–10) at one of three public secondary schools. This age range was selected for methodological purposes, making it possible to form two groups, the 12- to 14-year-olds (*n* = 207), corresponding to the first stage of adolescence (puberty, or early adolescence), and the 15- to 17-year-olds (*n* = 121), corresponding to the second stage of adolescence (adolescence *per se*). The final sample size from which all measurements were taken contained 328 subjects. Of these, 178 were male (54.3% of the sample) and 150 were female (45.7% of the sample). The mean age of the sample was 13.82 years, with a standard deviation de 1.19.

### Instruments

#### Conceptual subcompetency

**Figure 1:**
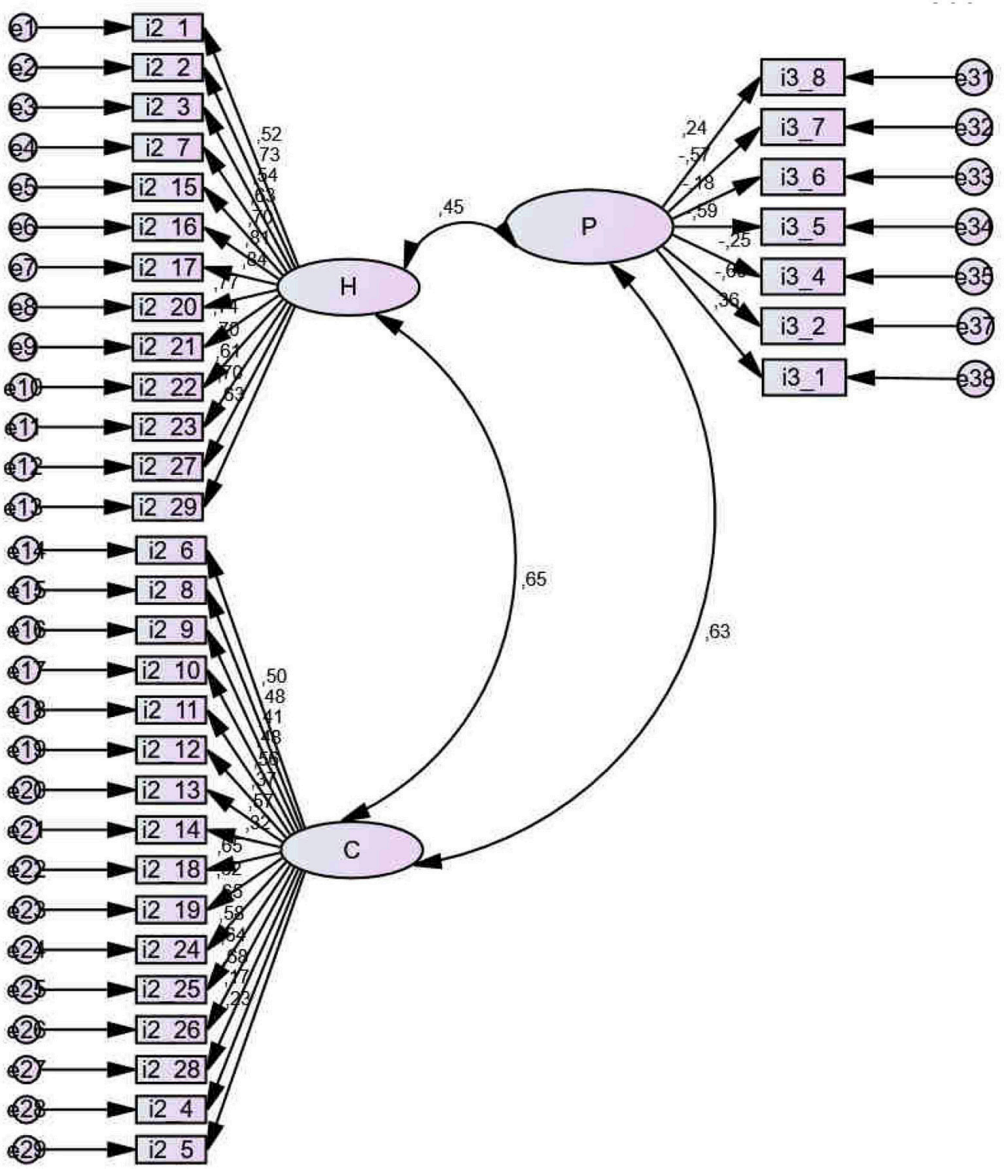
Confirmatory Factor Analysis (CFA) of EHCP scale. H, Facts; C, Concepts; P, Principles.

The scale *Evaluación de los Hechos, Conceptos y Principios sobre el Alcohol, EHCP* [Assessment of facts, concepts and principles about alcohol, AFCP] was used (Cubero and Sánchez, 2009a). The scale is composed of 38 items concerning the effects of alcohol use; psychometric analyses of this scale show reliability (alpha = 0.827) and consistent construct validity, with three factors: knowledge of facts, concepts, and principles concerning alcohol. Exploratory Factor Analysis (EFA) showed KMO = 0.801; Bartlett's Sphericity Test (*df* = 703) = 2767.595; *p* < 0.001. Confirmatory Factor Analysis (CFA) showed adequate indicators for the Default model: χ^2^ = 1612.957, *Degrees of freedom* (779–117): 662, *p* < 0.001; all the variances are significant for *p* < 0.001; NFI = 0.865; RFI = 0.848; IFI = 0.914; TLI = 0.902; CFI = 0.913; RMSEA = 0.025; HOELTER model = 1095 (*p* < 0.05),1138 (*p* < 0.01). See Figure 1 and Appendix A.

#### Procedural subcompetency

**Figure 2:**
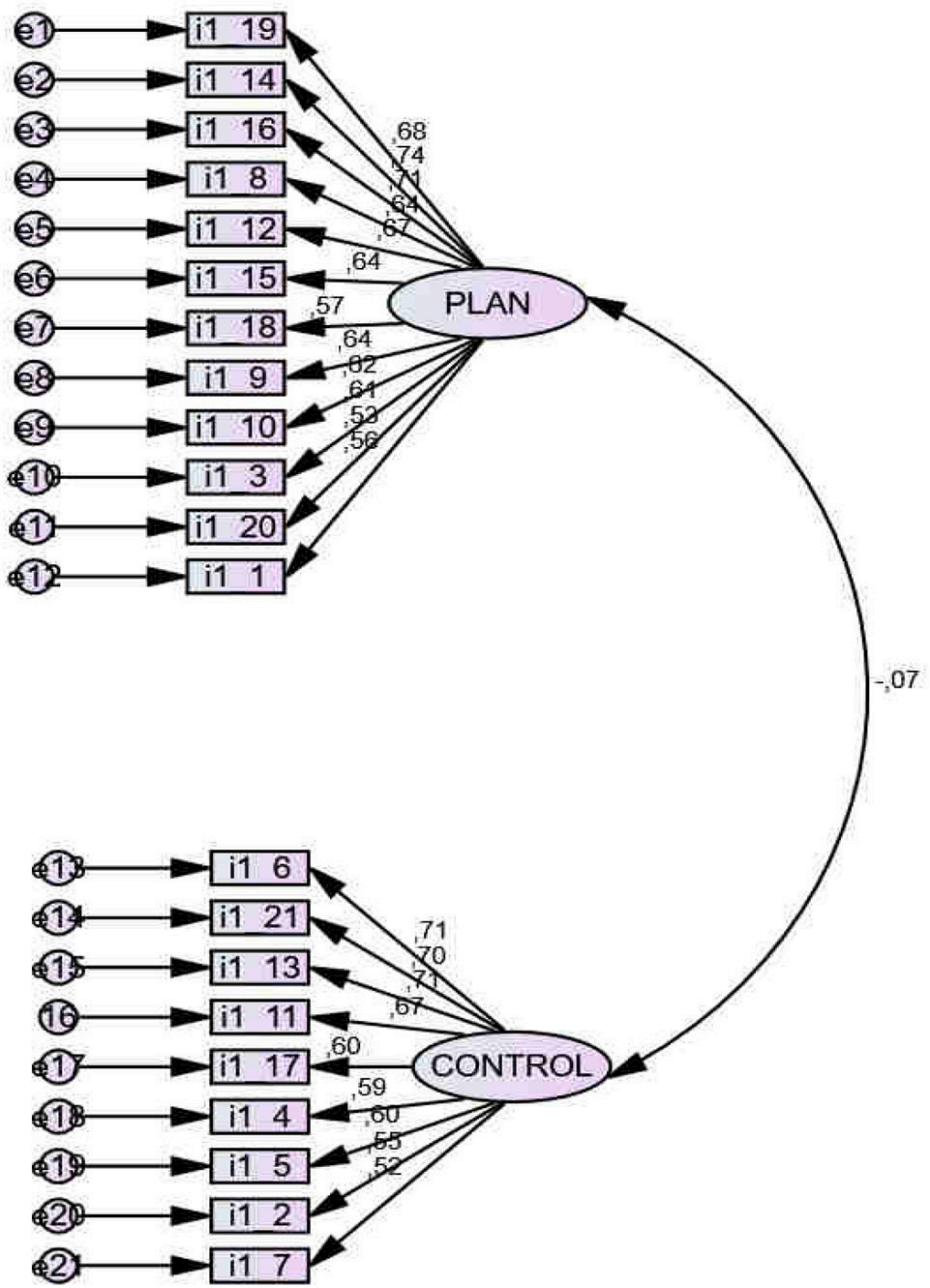
Confirmatory Factor Analysis (CFA) of the SRQ-21 scale.

The *SRQ, Self-Regulation Questionnaire* (Brown et al., 1999) was used, in its 21-item abbreviated Spanish version, SRQ-21 (de la Fuente, 2010). Its reliability (alpha = 0.826) and validity values are consistent, with two dimensions, planning and action control. *Exploratory Factor Analysis* (EFA) showed an index KMO = 0.985; Bartlett's Sphericity Test (*df* = 210) = 3603.882; *p* < 0.001. *Confirmatory Factor Analysis* (CFA) showed adequate indicators for the Default model: *Chi-square* = 408.448, Degrees of freedom (252–64):188, *p* < 0.001; all the variances are significant for *p* < 0.001; NFI = 0.894; RFI = 0.870; IFI = 0.940; TLI = 0.925; CFI = 0.9393; RMSEA = 0.022. However, this structure does not concur with others found in other samples (Artuch-Garde et al., 2017; Pichardo et al., in review). See Figure 2 and Appendix A.

#### Attitudinal subcompetency

**Figure 3:**
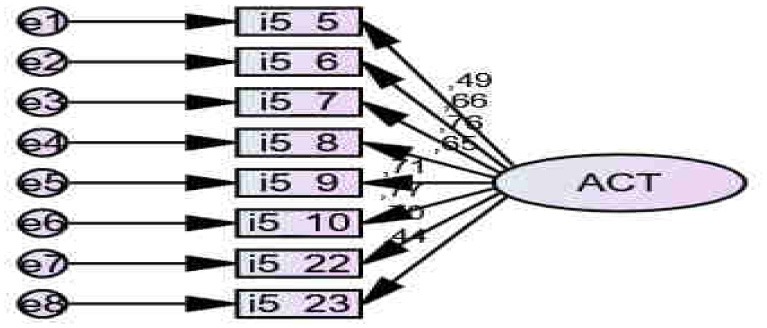
Confirmatory Factor Analysis (CFA) of Attitudes scale.

The scale for *Evaluación de las Actitudes ante el alcohol, EAA* [Assessment of Attitudes toward Alcohol, AAA] was used (Cubero and Sánchez, 2009b). A total of eight items assess attitudes and values toward alcohol (Alpha = 0.845). Exploratory Factor Analysis (EFA) showed KMO = 0.859; Bartlett's Sphericity Test (*df* = 28) = 529.335, *p* < 0.001. Confirmatory Factor Analysis (CFA) showed adequate indicators for the Default model: Chi-square = 58.574, Degrees of freedom (44–24): 20, *p* < 0.001; all the variances are significant for *p* < *0*.001; NFI = 0.903; RFI = 0.826; IFI = 0.34; TLI = 0.8; CFI = 0.2; RMSEA = 0.0; HOELTER model = 1349. See Figure 3 and Appendix A.

#### Adjusted behavior in interacting with alcohol

We used the Escala de Ajuste en la interacción con el alcohol [Scale of Adjustment in interacting with alcohol], which contains four items (Alpha = 0.915). This scale belongs to the Inventario de Evaluación de conocimientos, actitudes e interacción con el alcohol (Cubero and Sánchez, 2009c) (Inventory for Assessment of knowledge, attitudes and interaction with alcohol). See Appendix A.

### Procedure

The *Alado Program* is an online program (de la Fuente et al., 2012), designed for adolescents, to work on the three levels of subcompetencies previously established in the model. The program provides assessment and intervention in different matters of learning. It may be used by students, teachers and parents, but in this case it was applied only to students. The Teacher's Guide and Instructions for Use have been published.

Data collection instruments were applied over the course of the school year 2009-10, within the framework of the *Alado Project of Excellence* (2007–2010), through an online utility created for this purpose (www.alado.es). Specifically, these data correspond to the project baseline, having been collected in September-October, 2008. Previously, cooperation had been requested from the Teacher Development Center, from the students' parents and from the School Board, for their participation. The project was approved by the University Bioethics Commission (University of Almería). The students participated voluntarily. The parents were informed in writing. As the participants in the Project were minors, both the parents and school administrators gave written informed consent for the study. All data was collected in accordance with the principles of the Psychologist's Deontological Code and the Spanish Data Protection Act.

### Design and data analyses

For this set of data, obtained at the program baseline, an *ex-post facto methodological* design was used. Relationships were measured just as they occurred in the natural environment, without any treatment, using inferential and structural analyses based on the Presage-Process-Product Model (Biggs, 2001), and applied to educational problem areas in students in compulsory secondary education. The variables selected were as follows:

1. *Presage* variables, manipulated by selection: age and year in school,
2. *Process* variables, referring to intake *prevention subcompetencies*,
  1. Conceptual subcompetency: Knowledge (facts, concepts and principles) related to alcohol intake.
  2. Procedural subcompetency: Personal self-regulation skills, referring to planning and behavioral action control.
  3. Attitudinal subcompetency: Attitudes and values toward alcohol intake.
3. *Product* variables, referring to *interaction with alcohol*,
  4. Adequate behavior in interaction with alcohol: no contact.

Inferential statistical analyses (multivariate analysis, ANOVAs) were carried out using SPSS (v. 23.0) for Windows. For levels of the independent variable self-regulation, cluster analysis was used, obtaining three levels: low, medium and high. AMOS (v. 23.0) for Windows was used for the structural validity analysis of each inventory and for constructing the structural prediction model. To interpret the CFA and SEM model fit, we focused on the comparative fit index (CFI) and the root mean square error of approximation (RMSEA). CFI values equal to or more than 0.90 and 0.95 respectively were taken to indicate acceptable and close fit to the data (McDonald and Marsh, 1990). RMSEA values equal to or below 0.05 and 0.08 were taken to indicate close and acceptable levels of fit, respectively (Jöreskog and Sörbom, 1993). Keith (2006) proposed the following educational research benchmarks for *direct effects* in the form of beta coefficients: less than 0.05 is considered too small to be meaningful, above 0.05 is small but meaningful, above 0.10 is moderate, and above 0.25 is large. For *indirect effects*, we use Kenny's (2012) definition of an indirect effect as the product of two effects; using Keith's benchmarks above, we propose an educationally meaningful, small indirect effect = 0.003, moderate = 0.01, and large = 0.06.

## Results

### Interdependence relationships

#### Effect of the level of conceptual competency (facts, concepts, principles) on the remaining variables: KNOWING

A main effect appeared, statistically significant, of the IV *level of conceptual competence or knowledge* [*F*_(8, 210)_ = 2.451 (Pillai), *p* < 0.01, *n*^2^ = 0.085, power = 0.897]. Partial effects were shown in *attitude* [*F*_(2, 110)_ = 5.974, *p* < 0.01, *n*^2^ = 0.100, power = 0.872; post = 3 > 2,1, *p* < 0.01] and in *adjustment in alcohol consumption* [*F*_(2, 2110)_ = 3.003, *p* < 0.05, *n*^2^ = 0.050, power = 0.576]. No significant effect appeared for the variables *planning* and *control* (components of self-regulation). See Table 1.

**Table 1:** Direct values (means and sd) of the IV low-medium-high level of *conceptual competency* on the remaining variables.

**DV**	**IV (Knowledge and principles)**			
	**Low *n* = 15**	**Medium *n* = 89**	**High *n* = 7**	***post-hoc* (sheffe test)**
Planning	3.02 (0.64)	2.75 (0.64)	3.00 (0.45)	
Control	3.50 (0.99)	3.81 (0.67)	3.85 (0.28)	
Attitudes	1.60 (0.38)	2.05 (0.16)	2.66 (0.24)	1 < 2^*^; 1 < 3^**^
Adjustment in alcohol use	1.60 (0.42)	1.34 (0.99)	1.82 (0.48)	

* p < 0.05;

** *p < 0.001*.

#### Effect of the level of procedural competency (self-regulation) on the remaining variables: KNOWING HOW

A statistically significant main effect appeared for the IV *level of self-regulation* [*F*_(10, 208)_ = 3.236 (Pillai), *p* < 0.001, *n*^2^ = 0.135, power = *0.9*87]. Partial effects were a significant effect of *level of self-regulation* on knowledge of *facts* [*F*_(2, 107)_ = 3.316, *p* < 0.05, *n*^2^ = 0.050, power = 0.617; post = 3 < 1, *p* < 0.05], on knowledge of *concepts* [*F*_(2, 107)_ = 3.160, *p* < 0.05, *n*^2^ = −056, power = 0.595; post = 3 < 1, *p* < 0.05], on knowledge of *principles* [*F*_(2, 107)_ = 4.162, *p* < 0.01, *n*^2^ = 0.072, power = 0.723; post = 3 > 1, *p* < 0.05] and on *attitudes* [*F*_(2, 107)_ = 10.825, *p* < 0.001, *n*^2^ = 0.168, power = 0.989; post = 3 > 1, *p* < 0.05]. Finally, a significant effect appeared in the variable *adjustment in alcohol use* [*F*_(2, 107)_ = 3.360, *p* < 0.05, *n*^2^ = 0.086, power = 0.689; post = 3 > 1, *p* < 0.05]. See Table 2.

**Table 2:** Direct values (means and sd) of the *low-medium-high level of procedural competency (self-regulation)* on the remaining variables.

**DV**	**IV (Self-regulation)**			
	**Low *n* = 38**	**Medium *n* = 45**	**High *n* = 27**	***Post-hoc* (sheffe test)**
Facts	1.24 (0.28)	1.21 (0.28)	1.08 (0.12)	1 > 3^*^
Concepts	1.56 (0.39)	1.53(0.35)	1.35 (0.30)	1 > 3^*^
Principles	1.94 (0.35)	2.12 (0.29)	2.99 (0.20)	3 > 1.2^*^
Attitudes	3.40 (1.06)	3.98 (0.72)	4.37 (0.70)	3 > 2 > 1^**^
Adjustment in alcohol use	1.50 (0.63)	1.35 (0.59)	1.36 (0.58)	

* p < 0.05;

** *p < 0.01*.

#### Effect of the level of attitudinal competency (attitudes) on the remaining variables: WANTING

A main effect appeared, statistically significant, in the IV *attitude level* [*F*_(12, 206)_ = 4.378 (Pillai), *p* < 0.001, *n*^2^ = 0.203, power = 1.00]. Partial effects showed a significant effect of the *level of attitude toward alcohol* on knowledge of *facts* [*F*_(2, 107)_ = 5.858, *p* < 0.01, *n*^2^ = 0.099, power = 0.865; post = 1,2 > 3, *p* < 0.05], on knowledge of *concepts* [*F*_(2, 107)_ = 3.419, *p* < 0.05, *n*^2^ = 0.060, power = 0.631; post = 1 > 3 *p* < 0.05], and on knowledge of *principles* [*F*_(2, 107)_ = 11.644, *p* < 0.001, *n*^2^ = 0.179, power = 0.993; post = 3,2 > 1, *p* < 0.01]. Also, the *level of attitude toward alcohol* appeared with a significant partial effect on the degree of *self-regulatory control* [*F*_(2, 107)_ = 8.835, *p* < 0.001, *n*^2^ = 0.142, power = 0.968; post = 3,2 > 1, *p* < 0.001]. Finally, a significant effect appeared in the variable *adjustment in alcohol use*, but without clear directionality [*F*_(2, 107)_ = 2.285, *p* < 0.06, *n*^2^ = 0.050, power = 0.543]. See Table 3.

**Table 3:** Direct values (means and sd) of the *low-medium-high level of attitudinal competency* on the remaining variables.

**DV**	**IV (Attitudes)**			
	**1. Low *n* = 14**	**2. Medium *n* = 45**	**3. High *n* = 51**	***Post-hoc* (sheffe test)**
Facts	1.33 (0.24)	1.24 (0.28)	1.11 (0.21)	1,2 > 3^*^
Concepts	1.70 (0.40)	1.51 (0.36)	1.43 (0.33)	1 > 3^*^
Principles	1.70 (0.39)	2.03 (0.25)	2.11 (0.26)	3,2 > 1^**^
Planning	2.94 (0.78)	2.87 (0.62)	2.69 (0.61)	
Control	2.12 (0.99)	3.76 (0.56)	3.91 (0.56)	3,2 > 1^***^
Adjustment in alcohol use	1.46 (0.56)	1.55 (0.73)	1.26 (0.45)	

* p < 0.05;

** *p < 0.01*,

*** *p < 0.001*.

#### Structural prediction relationships

The results of structural analysis or pathway analysis (SEM) showed an acceptable model of relationships between variables (model 3). The relationship parameters of both models are set out below. See Table 4.

**Table 4:** Structural Models or pathway analysis (SEM).

**Model χ^2^**	**DF**	***P***	**NFI**	**RFI**	**IFI**	**TLI**	**CFI**	**RMSEA**	**HOELT (*p* < 0.01)**
1.3127.729 (1848-163)	1321	0.001	0.800	0.783	0.874	0.862	0.873	0.020	1161
2.3374.639 (2015-196)	1819	0.001	0.773	0.759	0.880	0.871	0.879	0.019	1440
3.4244.870 (2484-214)	2270	0.001	0.911	0.897	0.933	0.895	0.932	0.012	1463

##### Standardized Direct Effects

This predictive linear model establishes that latent variable Knowledge of *facts* (F) was a significant positive predictor (0.63) of the latent variable Knowledge of *concepts* (C), and this variable is positively predicted (0.63) for latent variable knowledge of *principles* (P). In addition, the facts (F) variable (0.63) and the principles variable (P) are positively predicted (0.28) of latent variable *interaction with alcohol* (INT). Complementarily, the latent variable *planning of behavior* (PL) was a significant predictor (0.36) of the latent variable attitudes (A). At the same time, *self-control of behavior* (CONTR) was a positively predictor (0.18) of latent variable *interaction with alcohol* (INT). Complementarily, the latent variable *attitudes* (A) predicted a positive and significant relationship (0.20) with the latent variable *interaction with alcohol* (INT). All the variances of errors were significant (*p* < 0.001). Table 5 shows the *direct effects* of the variables inherent in the model.

**Table 5:** Standardized Direct Effects (Default model).

	**F**	**C**	**P**	**PLAN**	**CONTR**	**ATT**	**INTERACT**
**FACTS**							
CONCEPT	0.647						
PRINCIPLES		0.680					
PLANNING							
CONTROL							
ATTITUDINAL							
INTERACTION	0.629		0.280		0.180	0.198	
**FACTS**							
I2_20	0.766						
I2_17	0.845						
I2_1	0.525						
I2_2	0.735						
I2_3	0.543						
I2_7	0.628						
I2_15	0.698						
I2_16	0.807						
I2_29	0.633						
I2_27	0.699						
I2_23	0.605						
I2_22	0.698						
I2_21	0.742						
**CONCEPTS**							
I2_5		0.228					
I2_4		0.175					
I2_28		0.676					
I2_26		0.638					
I2_25		0.582					
I2_24		0.646					
I2_19		0.622					
I2_18		0.652					
I2_6		0.503					
I2_8		0.476					
I2_9		0.408					
I2_10		0.476					
I2_11		0.564					
I2_12		0.370					
I2_13		0.568					
I2_14		0.325					
**PRINCIPLES**							
I3_1			0.402				
I3_2			−0.585				
I3_3			0.611				
I3_4			−0.238				
I3_5			−0.618				
I3_6			−0.186				
I3_7			−0.564				
I3_8			0.270				
**PLANNING**							
I1_19				0.681			
I1_14				0.736			
I1_16				0.714			
I1_8				0.634			
I1_12				0.670			
I1_15				0.636			
I1_18				0.573			
I1_9				0.637			
I1_10				0.624			
I1_3				0.611			
I1_20				0.534			
I1_1				0.560			
**SELF-CONTROL**							
I1_6					0.711		
I1_21					0.704		
I1_13					0.706		
I1_11					0.669		
I1_17					0.604		
I1_4					0.596		
I1_5					0.598		
I1_2					0.555		
I1_7					0.520		
**ATTITUDES**							
I5_23						0.451	
I5_22						0.702	
I5_5						0.504	
I5_6						0.666	
I5_7						0.766	
I5_8						0.650	
I5_9						0.703	
I5_10						0.755	
**INTERACTION**							
I5_1							0.728
I5_2							0.744
I5_3							0.709
I5_4							0.909

##### Standardized Indirect Effects

The model also contributed the existence of *multiple indirect* predictions among the variables. This predictive linear model establishes that latent variable knowledge of *facts* (F) was a positive significant predictor (0.440) of the latent variable knowledge of *principles* (P), and a positive predictor (0.124) of latent variable interaction with alcohol (INT). The latent variable knowledge of *concepts* was a positive predictor (0.192) of the latent variable interaction with alcohol (INT). The latent variable knowledge of *principles* was a positive predictor (0.072) of the latent variable interaction with alcohol (INT).

The latent variable knowledge of *facts* (F) was another indirect, positive predictor of items of C, P, and INT; the latent variable knowledge of *concepts* (C) was an indirect, positive predictor of items of C, P, and INT; the latent variable knowledge of principles (P) was indirect, positive predictor of items of INT.

The procedural and latent variable *planning* of behavior (P) was an indirect, positive predictor of items of *attitudes* (A) and *interaction* with alcohol (INT); the procedural and latent variable *self-control* of behavior (CONTR) was an indirect, positive predictor of items of *interaction* with alcohol (INT).

Complementarily, the attitudinal and latent variable *attitudes* toward alcohol (A) was an indirect, positive predictor of items of *interaction* with alcohol (INT). See Table 6.

**Table 6:** Standardized Indirect Effects (Default model).

	**F**	**C**	**P**	**PLAN**	**CONTR**	**ATT**	**INTERACT**
**FACTS**							
CONCEPTS							
PRINCIPLES	0.440						
PLANNING							
SELF-CONTROL							
ATTITUDES							
INTERACTION	0.124	0.192	0.72				
**FACTS**							
I2_20							
I2_17							
I2_1							
I2_2							
I2_3							
I2_7							
I2_15							
I2_16							
I2_29							
I2_27							
I2_23							
I2_22							
I2_21							
**CONCEPTS**							
I2_6		0.325					
I2_8		0.308					
I2_9		0.264					
I2_10		0.308					
I2_11		0.365					
I2_12		0.240					
I2_13		0.368					
I2_14		0.210					
**PRINCIPLES**							
I3_1		0.177	0.273				
I3_2		−0.257	−0.397				
I3_4		−0.105	0.162				
I3_5		−0.272	−0.420				
I3_6		−0.082	−0.126				
I3_7		−0.248	−0.383				
I3_8		0.119	0.183				
**PLANNING**							
I1_19							
I1_14							
I1_16							
I1_8							
I1_12							
I1_15							
I1_18							
I1_9							
I1_10							
I1_3							
I1_20							
I1_1							
**SELF-CONTROL**							
I1_6							
I1_21							
I1_13							
I1_11							
I1_17							
I1_4							
I1_5							
I1_2							
I1_7							
**ATTITUDES**							
I5_23				0.164			
I5_22				0.255			
I5_5				0.184			
I5_6				0.243			
I5_7				0.279			
I5_8				0.237			
I5_9				0.256			
I5_10				0.275			
**INTERACTION**							
I5_1	0.548	0.140	0.206	0.52	0.131	0.144	
I5_2	0.560	0.143	0.210	0.054	0.134	0.147	
I5_3	0.534	0.136	0.200	0.051	0.128	0.140	
I5_4	0.685	0.175	0.257	0.065	0.164	0.180	

##### Graphic representation of the structural model

**Figure 4:**
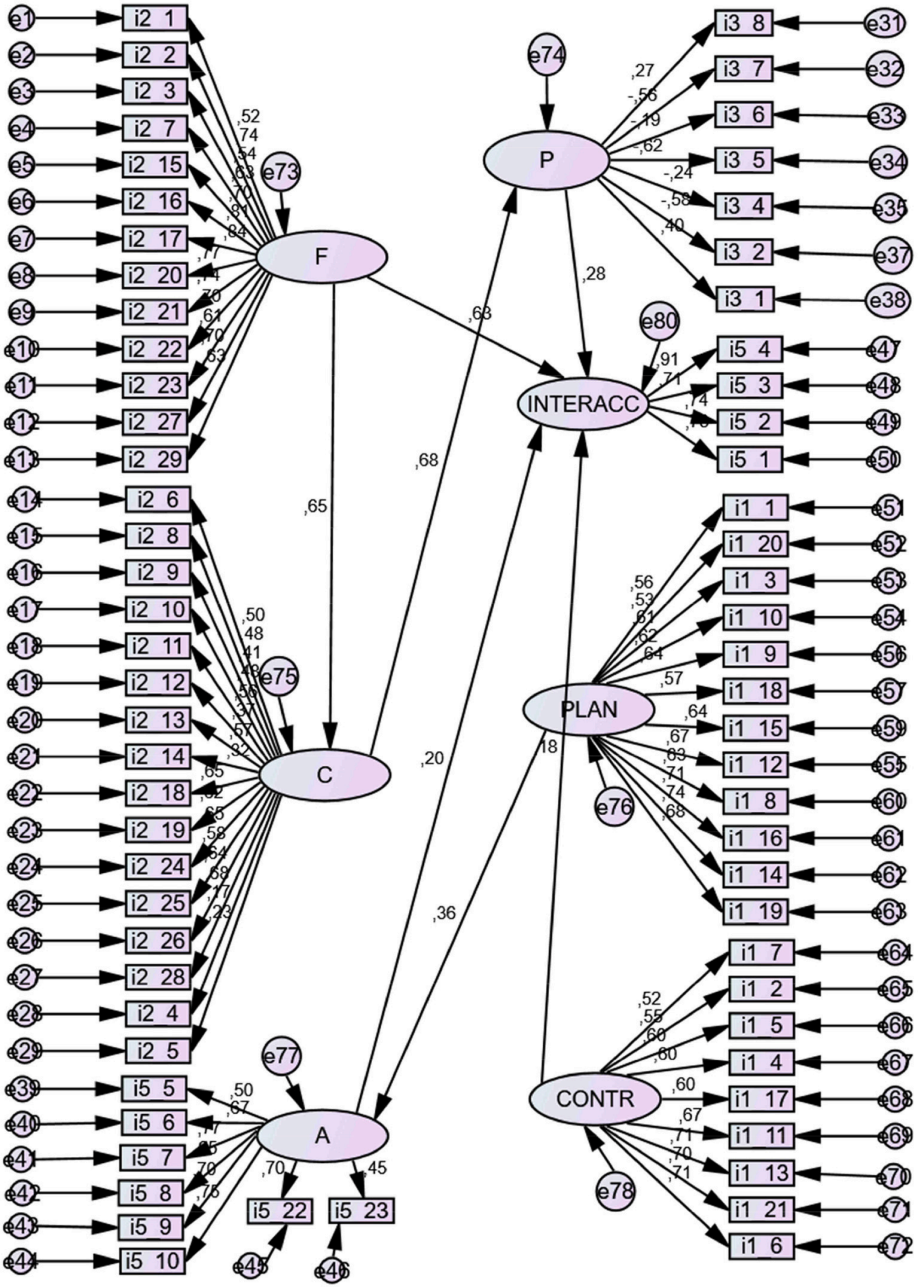
Graphic representation of the structural model. F, Facts; C, Concepts; P, Principles; PLAN: Planification; CONTR: Control; A: Attitudes: INTERACC: interaction with alcohol.

The final model is graphically represented in Figure 4.

## Discussion and conclusions

The *first objective* of this study was to determine any interdependence relationships between the low-medium-high level of each subcompetency with respect to the other subcompetencies, and to adjustment in interacting with alcohol. Thus, *Hypothesis 1* established that the low-medium-high level of *conceptual* knowledge (facts, concepts and especially principles), *procedural* knowledge (self-regulation, with self-control having greater weight) and *attitudinal* knowledge (values about alcohol intake) would be mutually determined, and that finally, these levels would determine the level of adjustment in interacting with alcohol.

This hypothesis was partially confirmed. Results provided evidence that the level of *conceptual competence* (facts, concepts, principles) determines the level of attitudes toward alcohol and the degree of adjustment in interacting with alcohol, that the level of *procedural competence* (self-regulation) determines the level of the principles and attitudes, and that the level of attitudinal competency (attitudes) differentially and inversely affects the level of knowledge about alcohol (facts and concepts), while directly affecting the degree of principles and of self-control. These results offer support for the competency model, by showing how each type of learning affects the others. One especially interesting result was that concept learning affected the degree of attitude acquisition, but not the degree of acquisition of self-control. However, the level of self-regulation did affect the learning of principles and attitudes toward alcohol. This represents more evidence on the importance of this behavioral level, corroborating that attitudes also affect the acquisition of principles and the degree of self-control. The second part of the hypothesis was also partially validated, showing that only the level of conceptual competence (principles) was accompanied by a higher level of adjustment. This result reinforces the idea that each variable analyzed, on its own, has a limited effect in determining the level of adjustment.

The *second objective* was to establish any structural prediction relationships between the different subcompetencies, with *Hypothesis 2* affirming that adjustment in interacting with alcohol (nonconsumption) would be determined both by conceptual type factors (more principles than facts/concepts), procedural type (self-regulation, especially level of control) and attitudinal type, although the latter are mediated by the procedural variables. In this case, the hypothesis was consistently fulfilled, with the appearance of a significant SEM model that showed different *direct effects*. Knowledge of facts affects concepts and these in turn affect the principles elaborated; knowledge of facts and of principles are the variables that most affect adjustment in interacting with alcohol. In addition, self-regulation affects adequate interaction via attitudes, and directly through self-control. Finally, attitudes also affect adjustment. However, the *indirect effects* are what has shown that all the levels of competencies—conceptual (facts, concepts and principles), procedural (planning and self-control) and attitudinal (attitudes)—are joint determinants in the degree of adjustment for nonconsumption of alcohol. These results are consistent with the prior evidence that has shown in part the importance of each of these variables (Moral and Ovejero, 2005; Moral et al., 2006).

From a behavioral point of view, both hypotheses show that different acquisitions are required in order to become competent in alcohol intake: (1) the acquisition of *knowing (cognitive level)*: to have assimilated the information about alcohol and its effects on the immature adolescent brain (Spear, 2015); (2) the acquisition of *wanting* to not drink (*motivational-affective level*), of attitudes and values (Foxcroft et al., 2015; O'Connor and Colder, 2015); (3) the acquisition of *knowing how (behavioral level)*: to have skills and meta-skills of self-regulated behavior (O'Connor and Colder, 2015; Yoon et al., 2015). This three-fold acquisition of integrated behavior, on the part of adolescents, having three different levels of complexity, has usually been addressed only partially and separately, from a psychosocial intervention approach.

From a complementary perspective, this investigation also makes a significant *methodological contribution*, with respect to the type of analyses carried out. Going beyond the usual descriptive or classic association strategies, a two-fold strategy was applied in order to validate the causal relations consistently, using inferential analysis and structural path analysis. The *inferential analysis* has shown clear interdependence relationships between the levels of the variables. *Path analysis*, based on the previous inferential analyses, has corroborated the multidirectionality of the proposed causations, producing an *empirical model* of this competency consistent with the one proposed, and offering a structural foundation for future interventions (MacCallum and Austin, 2000; Asparouhov and Muthén, 2014).

### Implications for educators and programs

This empirical evidence suggests different *implications for educators* who wish to make students competent, by working in primary prevention: (1) The concept of competence is *multidimensional* (Gagné, 2013); therefore, teaching and learning should work on all the levels of learning involved. (2) While information about alcohol is important–especially information that helps students construct an integrated, explanatory model of the effects of alcohol–excess information and inadequate modeling at a young age can be counterpreventive, with a dysregulatory effect. One notable effect was the inverse predictive relationship between adolescents' level of self-regulation and their degree of knowledge about alcohol. From the assumptions of the SRL vs. ERL Theory, this could be interpreted as an effect of a regulatory vs. dysregulatory context. In other words, adolescents who are growing up in a *regulatory educational context* (urban school and parents with secondary education) are trained to a higher level of self-regulation and they are protected from environments with excessive information about alcohol. The opposite would occur in *dysregulatory educational contexts* (marginalized school with compensatory education), where adolescents are allowed to have more direct contact with alcohol consumption, and early alcohol intake, or dysregulation, is encouraged. These results are consistent with the effect of the educational context at school, as seen in a previous dissertation report (Marcos, 2013), but research should continue to investigate this in more depth. (3) In addition, this evidence reaffirms the role and importance of planning for adolescents to learn self-regulation and self-discipline (Duckworth et al., 2011) – in association with proactive attitudes of substance rejection, since such attitudes are decisive to whether self-regulation and self-discipline will be practiced. Therefore, from the educational point of view of primary prevention, the experiences, information, modeling and attitudes offered by the educational context (family, school, peers) are determinants in encouraging regulation, nonregulation or dysregulation of the adolescent's alcohol intake (de la Fuente, 2017).

These results corroborate the idea that *educational programs* focusing on the primary prevention of alcohol intake should rest on two basic pillars: (1) an eminently *informational* thrust, for learning facts and concepts about the problem (also containing a developmental component); and (2) the more *developmental* thrust, referring to learning behavioral principles, behavior management skills or self-regulation, and the motivational or attitudinal aspect. *Conceptual, procedural* and competence would be impregnated with a holistic model of education, approaching each level of competency as different and needing to be approached differently. This question therefore deserves some profound analysis. In the first place, one must reinforce the importance of *principles* in alcohol use—norms, standards and goals for behavior (Pons et al., 1995). Without behavioral principles, we cannot determine the direction of self-regulation. It is therefore unadvisable to move toward preventive programs that have a large amount of merely conceptual information; rather, principles of behavior should be emphasized, as representations of reality and of oneself, especially in the stage of adolescence and pre-adolescence. In second place, training in *self-regulatory* behaviors—as a procedural meta-skill—is a must in educational programs for primary prevention of alcohol intake, given that this behavioral level has a clear effect on adjustment, as has been demonstrated in prior evidence from secondary and tertiary therapeutic programs (Fernández et al., 2009). Finally, there is no substitute for educational treatment of the attitudinal level. Attitudes must involve a desire for healthful behavior (Fergus and Zimmerman, 2005; García del Castillo and Días, 2007).

## Limitations and prospects

This study is not free of *limitations*. First, the effect of gender and of year in school were not analyzed, in an effort to streamline the design and show the relationships presented here, given that these effects were reported in an earlier research report (Marcos, 2013). However, the most important limitation is the absence of any analysis of the students' contextual variables (Smith et al., 2013) as is explained in the recent *Theory of Self-* vs. *External-Regulation*™ (de la Fuente, 2015, 2017). Without an interactive view of this problem, we obtain only a partial, personalist view. Future research should take into account the context, be it regulatory, nonregulatory or dysregulatory, in order to consider its likely impact in interaction with each adolescent's competency level (Leal, 2004; Márquez, 2006).

In the future, it would be desirable to perform research that incorporates the effect of other variables that recent research has identified as important in managing stress—an important aspect in adolescence, with all its personal, academic and social changes (González et al., 2002; Gómez-Fraguela et al., 2006). Likewise, longitudinal studies are needed, for prevention and for assessments (Arco and Fernández, 2002; Oliva et al., 2008). This report comes alongside many others that are presently being carried out with regard to adolescence (Casas, 2000; Ruiz-Aranda et al., 2006). Similarly, self-regulation is also the object of much current interest, as a psychological variable that is inherent to personal development competencies in subjects. Results obtained here indicate that it is worthwhile to continue down this road, given the power of this variable, in order that it may be given priority in planning future interventions (de la Fuente et al., 2009; Artuch-Garde et al., 2017).

## Author contributions

JdlF: Research design, data analysis and results writing; IC: Coordinator of the R & D Project and the Program; MS: Research of the R & D Project and the Program; FP: Program implementation and data collection; AG: Manuscript Review and Writing; JF: Writing and review in English.

### Conflict of interest statement

The authors declare that the research was conducted in the absence of any commercial or financial relationships that could be construed as a potential conflict of interest.
