# Supplementary material for: The Adolescent's Competency for Interacting with Alcohol as a Determinant of Intake: The Role of Self-Regulation
Source: Front Psychol. 2017 Oct 26;8:1800. doi: 10.3389/fpsyg.2017.01800 (PMC5662636; doi:10.3389/fpsyg.2017.01800)
Supplement: Supplementary file 1 [file DataSheet1.docx]

APPENDIX A

*SRQ-21. Spanish adaptation* (de la Fuente, 2010)

_______________________________________________________________________________________

I1_1. I usually keep track of my progress toward meeting my goals.

I1_2. I don’t notice the effects of my actions until it’s too late (-)

I1_3. I’m able to meet the goals I set for myself

I1_4. I put off making any decisions (-)

I1_5. It’s hard for me to realize when I have had enough of something (alcohol, food, candy, etc.) (-)

I1_6. When I have decided to do something, it’s hard for me to stick with it until the end (-)

I1_7. I don’t seem to learn from my mistakes (-)

I1_8. Usually after making a mistake just once, I’ve already learned my lesson

I1_9. I have my personal convictions and I try to live up to them

I1_10. As soon as I see a problem or a challenge, I start looking for possible solutions.

I1_11. It’s hard for me to set goals (-)

I1_12. When I am working toward some kind of change, I pay a lot of attention to how it’s going

I1_13. I find it hard to make plans to reach my goals (-)

I1_14. I set goals and keep track of my progress

I1_15. I can usually find several different possibilities when I want to change something

I1_16. If I make up my mind to do something, I pay a lot of attention to how it’s going

I1_17. I often don’t realize what I am doing until someone points it out to me (-)

I1_18. I usually think before I act

I1_19. I learn from my mistakes

I1_20. I know what I want to be like

I1_21. I give up easily (-)

*Assessment of facts, concepts and principles about alcohol, AFCPA (Cubero & Martínez, 2009a)*

_______________________________________________________________________________________

I2_1. Is alcohol a drug?

I2_ 2. Does each type of beverage have different alcohol content?

I2_ 3. If an alcoholic drink has higher proof, does that mean higher alcohol content?

I2_ 4. Does drinking two mugs of beer amount to more alcohol consumption than drinking a whisky?

I2_ 5. Does it take about 30 minutes for your body to eliminate one serving of alcohol (e.g. a beer)?

I2_6. Is alcohol eliminated from your body more quickly if you are used to drinking?

I2_7. Once alcohol is in the bloodstream, can it spread to the whole body and reach all of our organs?

I2_8. If adults have wine or beer every day with meals, does that have the same effect as my drinking it all at once on the weekend?

I2_9. Alcohol is harmful to your body and your brain only when you overdo it and end up passing out.

I2_10. When adults say that if you drink a lot one day, you can die from respiratory arrest, they are trying to scare us, but it is not true. A single drinking binge cannot kill you.

I2_11. If a friend starts to feel bad, or partly loses consciousness after drinking a lot, the best thing is for them to vomit it all and then rest till they feel better. It is not necessary to call an ambulance.

I2_12. It does not matter how much alcohol a person consumes. The only important thing for alcohol to not produce long-term consequences is not getting drunk.

I2_13. The only reason teenagers cannot drink alcohol is because it is against the law.

I2_14. If you get drunk it does not matter, a cold shower or strong coffee will clear your head.

I2_15. Can alcohol consumption lead to unsafe sexual practices?

I2_16. Does starting to drink as a teenager increase the chances of becoming alcoholic as an adult?

I2_ 17. Does alcohol affect how your brain functions?

I2_18. If I only drink alcohol on the weekends, it does not hurt my body.

I2_ 19. If I drink a large amount of alcohol, but drink it very quickly, it will not be as dangerous as drinking it slowly.

I2_ 20. Alcohol kills many brain cells.

I2_21. Alcohol does more harm to a teenager’s brain than to an adult’s.

I2_22. Continual alcohol use on the weekends can bring about memory problems and failures in my studies.

I2_23. Alcohol enters the brain soon after drinking and alters its functioning.

I2_ 24. Some of my friends are used to drinking alcohol and they no longer get drunk so quickly. This means that alcohol does less damage to their brain and body.

I2_ 25. If you hold your alcohol well, you drink a lot and do not get drunk, it is because your brain and body have gotten used to it and it no longer has as many negative effects on your brain.

I2_26. If a teenager only drinks at the usual weekend bash, he or she will not get alcohol-produced disease. That only happens if you drink a large amount every day.

I2_27. Alcohol use on the weekends can eventually cause disease of the heart, liver or digestive system.

I2_ 28. The human brain is perfectly formed at the age of 12. It no longer has to change, so we can drink after that age without fear of brain damage.

I2_ 29. The teenage brain is immature and must continue to develop until at least the age of 20.

I3_1. Which of the following is a short-term effect of alcohol use?

-You lose coordination and your reasoning ability declines+++

-Your body retains water and you feel bloated

-You skin breaks out and you get a rash

I3_1. Which of the following is an effect of alcohol abuse over a period of time?

-Weight loss and muscle wasting

-Low blood pressure

-Liver disease such as cirrhosis or cancer+++

I3_3. What is the best method for relieving a hangover?

-Drink water, because it helps rehydrate the body+++

-Take a cold shower, because it stimulates your circulation

-Have some strong coffee, because it makes your heart pump

I3_4. How can you tell how much alcohol is in a drink?

-By its volume: a larger amount means more alcohol

-By the number of grams of alcohol it contains (a standard drinking unit (SDU) contains 10g)+++

-By its weight: a greater weight means more alcohol

I3_5. Which of these drinks contains more alcohol?

-a 330ml can of beer

-a 100ml glass of wine (red or white)

-a shot of distilled liquor (e.g. rum, gin or vodka)+++

I3_6. At what age can a young person legally buy and consume alcohol in Spain?

-16 years

-18 years+++

-21 years

I3_7. Alcohol and driving. In Spain:

-It is illegal to consume more than two alcoholic beverages when driving

-It is illegal to drive after consuming alcohol if you have not had anything to eat

-It is illegal to drive with a blood alcohol concentration of more than 0.5g/l (grams per liter) +++

I3_8. When must adults totally abstain from alcohol use?

-If they are going to drive, use machinery or work at a height+++

-If they are more than 40 years old

-If they are going to ride as passengers in a car

I3_9. Why does alcohol affect men and women differently?

-A woman’s liver metabolizes alcohol more quickly

-Because a woman’s body has a higher fat index and less water, the blood alcohol concentration is higher+++

-A man’s stomach is larger, so the alcohol is absorbed more slowly

*Inventory for Assessment of Attitudes and Interaction with Alcohol, AAIA (Cubero & Sánchez, 2009b, 2009c)*

______________________________________________________________________________________

*Interaction*

I5_1. How often do you consume an alcoholic beverage?

I5_2. How often over the past year were you unable to stop drinking once you had started?

I5_3. How often over the past year did you feel regret or feel guilty after drinking?

I5_4. Has a family member, friend, doctor or healthcare professional expressed concern about your consumption of alcoholic beverages, or suggested that you stop drinking?

*Attitudes*

I5_5. Small quantities of alcohol are harmful

I5_6. Alcohol is one of the ills of our society

I5_7. I dislike people drinking alcohol.

I5_8. I admire a person who does not drink alcohol.

I5_9. I am not curious about trying alcohol

I5_10. I would reject an invitation to drink alcohol
